# Evaluation of surveillance system and response for maternal and perinatal mortalities in Meda Welabu District, Ethiopia, 2023

**DOI:** 10.11604/pamj.2024.47.133.42963

**Published:** 2024-03-22

**Authors:** Eshetu Zemen, Yimer Seid Yimer, Negussie Deyessa, Yonas Abebe

**Affiliations:** 1 Department of Field Epidemiology, Field Epidemiology Training Program, Addis Ababa University, Addis Ababa, Ethiopia,; 2 Department of Epidemiology and Biostatistics, School of Public Health, Addis Ababa University, Addis Ababa, Ethiopia,; 3 Ethiopia Field Epidemiology Training Program Field, Federal Ministry of Health Ethiopia, Addis Ababa, Ethiopia

**Keywords:** Surveillance system evaluation, maternal and perinatal death surveillance, Meda Welabu Woreda, Oromia, Ethiopia

## Abstract

The weekly disease surveillance system (WDSS) serves as a precursor to possible public health emergencies. The Meda Welabu Woreda Bale Zone in Ethiopia has reporting rates of 87% overall timeliness and 88% completeness in 2023, falling short of the 100% objective. Low reporting rates could mean that epidemics in the province are only discovered later. In the Meda Welabu Woreda Bale Zone of Ethiopia, the study was carried out to assess the WDSS maternal and perinatal death surveillance response (MPDSR). Using the most recent Centers for Disease Control (CDC) criteria for assessing public health monitoring systems, we carried out a descriptive cross-sectional analysis. Data from the health workers were gathered through key informant interviews and questionnaires given by the interviewer. Using checklists, the availability of resources was evaluated. Twenty-two health personnel and twelve Health Extension Workers were questioned; of them, 15 (44%) were females. Nurses made up 18 (53%) of the health personnel. Only sixteen (47%) of the respondents were aware of the WDSS goals, compared to thirty-four (53%) who were aware of the deadlines for submitting data to the next level. A total of eight (24%) responders received training in using the WDSS. 26(76%) respondents said they would be willing to continue participating in the WDSS, whereas 6 (18%) respondents said they had analyzed the data from the WDSS. Of the health facilities, seven (50%) reported having issues with the district public health emergency officer. However, low attention to immediately report on maternal and perinatal death (42.9%). It was concluded that the WDSS was adaptable, reasonable, and easy to use. That was erratic and premature, though. We suggest that healthcare professionals in the province receive training on maternal and perinatal death surveillance response. In Meda Welabu Woreda Bale Zone conducted an evaluation in 2023 of the weekly disease surveillance system, maternal and perinatal death surveillance response. Launched in 1998, the system tracks weekly trends of diseases under surveillance to provide an early warning of any dangers to public health, but maternal and perinatal death surveillance were included on 2013. On the other hand, in 2023, the overall completion and timeliness of reports was 88%, falling short of the 100% aim. Low rates of reporting could mean that outbreaks and quality of service in the province were discovered later than expected. Using current centers for disease control criteria and interviewer-administered data, a descriptive cross-sectional study was undertaken.

## Introduction

The maternal death surveillance and response system was introduced in 2013 to produce contextual knowledge on the burden and distribution of current causes and contributing factors for programmatic and individual-level decision-making and the inclusion of Maternal Death Surveillance and Response (MDSR) in a country´s health strategy is considered a key component of efforts to reduce maternal mortality [1]. One significant quality-of-care quality of care (QoC) initiative that tries to enhance maternal and newborn health (MNH) is Maternal and perinatal death surveillance and response (MPDSR) [2]. Assessing all deaths of women of reproductive age (WRA) and identifying those that happened during a woman's pregnancy or within 42 days after the end of a pregnancy (suspected maternal death) is the first step in detecting maternal fatalities. If a WRA passes away in a medical facility, her medical file should be reviewed to find out whether she was pregnant. Community health workers (CHW), traditional birth attendants, or other community leaders may report suspected maternal fatalities; verbal autopsies should then be carried out to ascertain the probable cause of death. Deaths at health facilities should be recognized and reported to the proper authorities within 24 hours, and deaths in communities should be reported to the authorities within 48 hours [3]. Public health surveillance is the ongoing, systematic collection, analysis, interpretation, and dissemination of data regarding a health and health-related event for use in public health action to reduce morbidity and mortality and to improve health. A well-functioning disease surveillance system is critical to the health system in providing evidence-based information for the planning, implementation, monitoring, and evaluation of public health intervention programs [4].

Since 2008 the MoH launched a reform and restructuring of the health sector into different core processes, in particular disease surveillance and response, with the concept of Business Process Re-engineering (BPR). This helps the surveillance of priority diseases to be a dependable system as public health emergency management (PHEM) center. Currently, there are 22 high-priority diseases and events concerning public importance, potential to epidemic, international concern, and diseases under eradication and elimination under surveillance. Of these, maternal death is one of the immediately reportable priority events (Table 1) [4]. Maternal death is the death of a woman while pregnant or within 42 days of the termination of pregnancy irrespective of the duration and site of the pregnancy, from any cause related to or aggravated by the pregnancy or its management, but not from accidental or incidental causes. The maternal death surveillance and response (MDSR) system is a continuous cycle of maternal death identification, notification, and review, followed by the interpretation of review findings, responses, and actions to prevent future deaths [3].

**Table 1:** list of weekly immediate and weekly re-portable diseases in Ethiopia

Immediately reportable disease/events	Weekly reportable disease/events
Acute flaccid paralysis	Dysentery
Anthrax	Malaria
Avian human influenza	Meningitis
Cholera	Relapsing
Dracunculiasis/Guinea worm	Typhoid fever
Measles	Typhus
Neonatal tetanus	Severe acute malnutrition
Pandemic influenza A (H1N1)	Others with new guideline 11
Rabies	-
Smallpox	-
SARS	-
Viral hemorrhagic fever (VHF)	-
Yellow fever	-
Maternal death	-
Perinatal death	-

Approximately 810 women die every day in the world due to preventable causes related to pregnancy or childbirth. More of these maternal deaths (94%) occur in developing countries, anymore than half in sub-Saharan Africa, including Ethiopia. The maternal mortality ratio in the least developed countries is as high as 415 per 100,000 births, versus 12 per 100, 000 in Europe and Northern America and 7 in Australia and New Zealand. There are large disparities between countries, with 11 countries having extremely high maternal mortality ratios of 600 or more per 100, 000 live births in 2017 [5]. According to the 2016 Ethiopian Demographic and Health Surveys (DHS), the maternal mortality ratio (MMR) is around 412/100,000 lbs [6]. Despite having made significant reductions in maternal mortalities during the last decades, Ethiopia continues to have a high estimated rate of maternal deaths. Most of these losses are believed to be preventable with high-quality, evidence-based interventions delivered before and during pregnancy, during labor, and in the crucial hours and days after birth [6]. Even though perinatal mortality has declined globally; it is still the major public health concern in sub-Saharan African countries. Ethiopia is one of the sub-Saharan countries which contribute the highest-burden of perinatal mortality, with a devastating rate in some of the regions.

Therefore, this study aimed to identify the determinants of perinatal mortality in the high mortality regions of Ethiopia [7]. Improving maternal health is one of the thirteen targets for Sustainable Development Goal 3 (SDG-3) on health adopted by the international community in 2015. Reducing maternal mortality and improving maternal health is a top priority of the Ethiopian Federal Ministry of Health (FMOH) as reflected in the Health Sector Transformation Plan. Federal Ministry of Health of Ethiopia aims to eliminate preventable maternal deaths and thus has been implementing maternal death surveillance and response since 2013. The system operates as part of the national public health emergency management (PHEM) system at the health facility and community level to identify and capture maternal deaths happening in both facilities and the community. Reporting maternal deaths through weekly surveillance lags behind the other reportable conditions within the surveillance system [8]. The formal flow of surveillance data is usually from reporting site to the next level up to the national level and World Health Organization (WHO).

**Figure 1:**
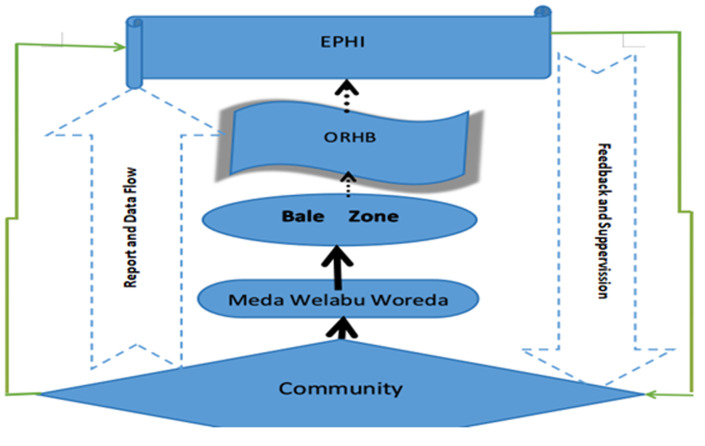
data flow framework structure Meda Welabu Woreda, Bale Zone, Ethiopia, 2023(10)

The community and health facilities at the lowest level, particularly Health posts are the main source of information about the occurrence of health-related events (Figure 1). The information collected from this site is compiled in standardized forms, analyzed, and then forwarded to the district health office by health centers. The district level compiles, analyzes, and sends the data to the zonal level by using a standard form. Similarly, the zonal level compiles and analyzes the report and sends the compiled data to the region by using a standard form and the national level receives the compiled data from the region. Feedback and information sharing follow the same route, but in the reverse direction [9]. Despite this, according to the MDSR 2019 annual report, only 10% of maternal deaths are captured by weekly notification, and 8% are captured by case-based reports, based on Ethiopia Demographic and Health Survey (EDHS) 2016 estimates of maternal deaths throughout the country. There were regional differences in reporting rates as compared to estimated numbers of maternal deaths. Dire Dawa city administration and Harari regions reported 52% and 42% of expected maternal deaths. Lower reporting rates were received from Ethiopia Somali and Southern nations nationalities and peoples (SNNP) regions, which were 0.1% and 4% respectively. Oromia region notified 13.3% of maternal deaths from estimation and from notified deaths, only 6.9% were reviewed and reported by case-based. From reviewed deaths, the Bale Zone accounts for 10.1% of the region and 3% of the national report [3].

This study is intended to evaluate the surveillance system in Meda Welabu district, mainly focusing on the surveillance system of maternal death surveillance and response activities. In the district, there were no maternal death surveillance system evaluations conducted before. The purpose of the evaluation is to monitor progress in the implementation and overall performance of the MDSR system. Therefore, specific indicators are identified based on the WHO surveillance evaluation to assess the structure, core, and support functions and quality of the MDSR system. The findings of this evaluation can be used as input to strengthen the overall surveillance system activities of the district to achieve its intended objectives and purpose. In Ethiopia, maternal mortality is still a significant public health issue. The readiness of the Meda Welabu Woreda health system to provide information for making decisions using the MPDSR system is described in this surveillance evaluation system. For this study aims to evaluate the maternal and perinatal death surveillance and response system in Meda Welabu Woreda, Bale Zone, Oromia, September 30, 2023.

## Methods

**Figure 2:**
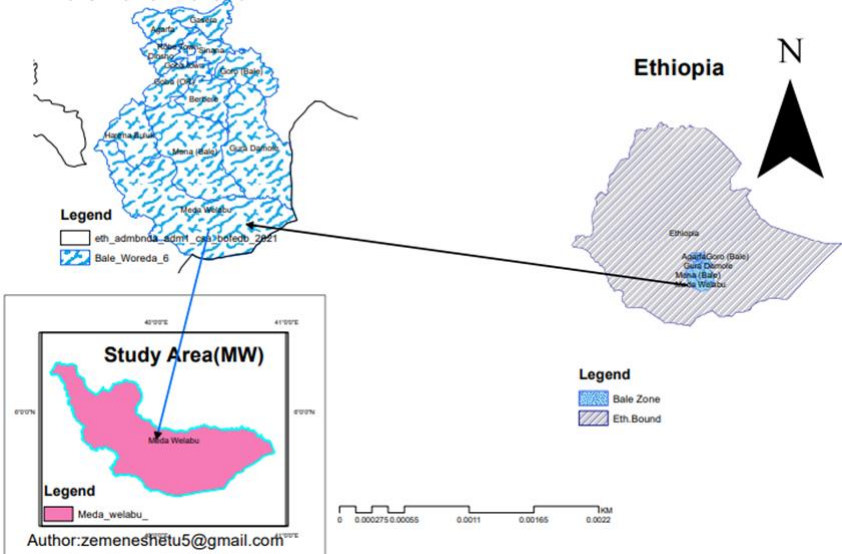
administrative map of Meda Welabu Woreda showing the area surveillance system evaluation done Bale Zone, Ethiopia, 2023

**Study area:** the study was carried out in the eighteen rural and two urban villages that make up the Meda Welabu Woreda Bale Zone in Ethiopia. Pastoralist is the main driver of the province's economy. It is located in southeast Ethiopia, bordering by East Borana and Guji zones as well as by Dalomena and Harena Buluk district in North east, Somali region on south-eastern part. The region has seen significant poverty as a result of droughts and a lack of economic possibilities, and internal displacement is a frequent outcome of conflict with the neighboring Somali region. According to the national population census data from 2008, the province is home to 186,000 people. There are six health centers, one government hospital, and twenty-five health posts in the province. The province's high risk of epidemic-prone diseases is a factor in the surveillance system's significance for public health (Figure 2).

**Study population:** the study populations were public health emergency management (PHEM) focal persons, the health center head and MCH coordinators in all health centers in the district, and health extension workers (HEWs) in sampled health posts. The WDSS reporting week begins on Monday at 8: 30 and ends on Sunday at 2400 hours. Every day, the nurses and Health Extension staff at the rural health center complete an illness registration form. This data is compiled by Monday and delivered via mail, phone, or hard copy to the district health public health emergency management unit. The district then compiles all the reports and, by Tuesday, submits them to the zonal public health emergency management officer.

**Study design and period:** the study conducted was a retrospective descriptive study, utilizing Weekly data surveillance data. The study was conducted from 1^st^ August to 30^th^ September 2023.

**Data collecting tools and procedures:** maternal and perinatal death surveillance and response were considered in the evaluation of the system. The study was evaluated using WHO surveillance evaluation and CDC framework, updated guidelines for evaluating public health surveillance systems. The data was collected through document observation, record review, and face-to-face interviews with key stakeholders.

For the quantitative data, the sources were surveillance reporting formats, records, and documents in health facilities. For the qualitative study, the key informants such as health centers, PHEM focal persons, MNCH coordinators, heads of health centers, and health extension workers from health posts were interviewed using a semi-structured interview guide with a flexible probing technique. Data regarding the availability of surveillance guidelines, reporting formats and documentation, registration of cases, completeness and timeliness of the report, and the quality of data will be assessed by a quantitative survey by examining report format, records, and documents.

Information concerning the structure and operation of surveillance and reporting systems, case detection, and attributes such as simplicity, flexibility, predictive value positive, Representativeness, and stability of the system were explored by the key informants. Information concerning the analysis and interpretation of surveillance data, epidemic preparedness, and management, supportive supervision and feedback, and the acceptability of surveillance systems were assessed both by document observation and explored by key informants, data were collected by investigators. In the Meda Welabu district, the primary health care unit (PHCU) is conducting surveillance on the population. The projected total population from the 2007 census is being monitored, along with an estimated percentage of pregnant women. Additionally, the expected maternal death rate and perinatal death rate from the EDHS 2016 data are being used for surveillance and investigation purposes (Table 2).

**Table 2:** the population under surveillance in Meda Welabu district by primary health care unit, 2023

S.N	Name of primary health care unit	Projected total population from the 2007 census	Estimated to be pregnant (3.47%)	Expected maternal death from Ethiopia Demographic and Health Survey 2016 (412/100,000LBs)	Expected perinatal death from Ethiopia Demographic and Health Survey 2016 (41/1000)
1	Bidire	28,821	1000	4	41
2	Mada	24,838	862	3	35
3	Gobele	23,997	833	3	34
4	Waduma	32,789	1138	5	47
5	Oborso	49,664	1723	7	71
6	Ware	26,587	926	4	38
7	Meda Welabu hospital	186,000	6478	26	266
8	Meda Welabu Woreda	186,000	6478	26	266

**Data processing and analysis:** the quantitative data were entered and analyzed using Microsoft Excel 2013. Quantitative findings were summarized by their frequency and proportion. Qualitative data were analyzed manually using thematic analysis. Data were cleaned before analysis, and the qualitative findings were narrated and summarized based on thematic areas to supplement the quantitative results.

**Operational definition:** terms used in the evaluation were operationally mentioned as follows.

**Reporting:** refers to the process by which surveillance data moves through the surveillance system from the point of generation. This study will be evaluated based on the availability of reporting channels and a report rate of 1-30 weeks in 2023.

**Stakeholders:** the organizations or individuals that generate or use surveillance data for the promotion of health, prevention, and control of diseases.

**Usefulness:** contribution to prevention and control of adverse health-related events. The level of usefulness was measured by the actions taken as a result of the analysis and interpretation of the data from the public health surveillance system, the system useful to detect diseases and outbreaks, and providing estimates of the magnitude of morbidity and mortality.

**Simplicity:** simplicity of structure and ease of operation. In this study, the simplicity of the surveillance system was measured in terms of clear and easily understandable case definition, route of data flow, difficulty in completing surveillance data, and time taken to complete surveillance data.

**Completeness:** measured by the proportion of health facilities that submitted a weekly report to the higher level, out of the expected facilities in the catchment area.

**Timeliness:** the timeliness of the report was assessed at two levels. First, it was calculated by assessing how many of its expected reports were submitted to the next level within the prescribed time. The report is timely for the health facilities if a weekly report is submitted to the district health office every Monday before midday. Second, the time interval between the onset of a health-related event and the reporting of the event, and the time required for the identification of an outbreak and initiation of control and prevention measures.

**Flexibility:** ability to adapt to changing information needs and operating conditions. In this study, the flexibility of the surveillance system was assessed in terms of accommodating changes in the existing procedure, a revised case definition, additional data sources, personnel, case detection, and reporting format.

**Data quality:** completeness and validity of recorded data. Completeness was examined by the number of cases and deaths, the date the report was sent and received, and the blank responses. Validity of recorded data at health posts compared to the reported data at health centers.

**Acceptability:** willingness of persons and organizations to participate. Acceptability was measured by completeness of report forms, timeliness of data reporting, and use of standard case definition.

**Positive predictive value:** the proportion of cases detected by the surveillance case definition that has the disease.

**Sensitivity:** proportion of true events detected by the system and ability to detect outbreaks. Representativeness: ability to describe events over time and their distribution by place and person. Measured in terms of distribution of a health-related event by time, place, and person, the health service coverage, and the reporting of surveillance data from all health facilities.

**Stability:** reliability and availability of surveillance systems. Measured by the availability of a surveillance focal person at all levels and integration of the system to routine healthcare delivery.

**Community case definition:** (probable maternal deaths): death of a woman of reproductive age (between 15-49 years of age).

**Suspected maternal deaths:** community case definition plus at least one of the following: i) died while pregnant; ii) died within 42 days of termination of pregnancy or; iii) missed her menses before she died.

**Standard case definition (confirmed maternal deaths):** the death of a woman while pregnant or within 42 days of the end of pregnancy (irrespective of the duration and site of pregnancy), from any cause related to or aggravated by the pregnancy or its management but not from accidental or incidental causes.

**Positive knowledge:** those respondents who achieve a median or higher on knowledge questions relating to the MPDSR are considered to have satisfactory knowledge [10].

**Positive attitude:** respondents who scored higher than the median on the questions assessing their attitudes regarding the MPDSR [10].

## Results

A total of 31 reporting units (24 health posts, 6 health centers, and 1 district hospital) submitted surveillance reports to the Meda Welabu district health office in 2023. A total of 14 of these sites were evaluated for this surveillance system evaluation. A woreda health office is among the six (100%) health centers, one hospital, and six (25%) health posts that make up this group. A total of 34 healthcare workers participated in the evaluation, with a majority being men among health professionals and all women among health extension workers. The age range and median age differed between the two groups. The majority of health workers were nurses, with varying years of experience. The facility reported 13 perinatal deaths and 16 maternal deaths during the evaluation (Table 3). The study's findings indicate that in 2023, the weekly surveillance report completion rates for health centers and health posts would be 88% and 87%, respectively. The district's overall completion rate for reports was 88%. The district's average percentage of timely surveillance reports was 82%.

**Table 3:** socio demographic characteristics of health professional and health extension workers in selected health facilities for surveillance evaluation facilities, 2023

Name of health facility	Respondent's characteristics		Frequency
Meda Welabu hospitals	Sex	Male	12
		Female	3
	Professional	Health officer	0
		Nurse	2
		Midwifery	13
	Education level	Degree	15
		Diploma	0
Meda Welabu Woreda health office	Sex	Male	2
		Female	0
	Professional	Health officer	0
		Nurse	2
		Midwifery	0
	Education level	Degree	2
		Diploma	0
Health center	Sex	Male	15
		Female	15
	Professional	Health officer	6
		Nurse	12
		Midwifery	12
	Education level	Degree	27
		Diploma	3

**Figure 3:**
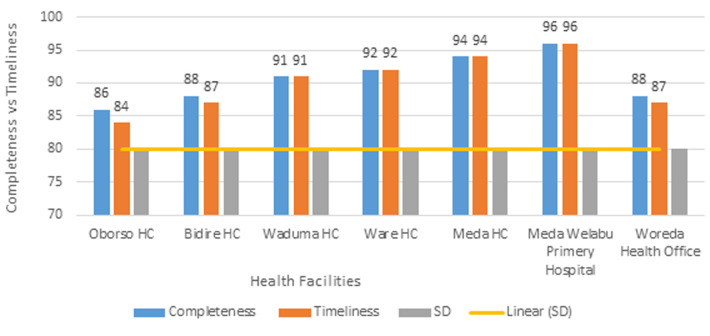
surveillance data completeness and timeliness, Meda Welabu Woreda Bale Zone, 2023

The normal healthcare delivery system is fully linked with the surveillance system, which has been put into place all year. The primary hospital report from Meda Welabu also scored 96% for timeliness and completion. However, there are notable gaps in the timeliness, accuracy, and completeness of the data, according to the surveillance system study carried out on maternal and perinatal death surveillance system (MPDSR) in Meda Welabu Woreda, Bale Zone. The results are summed up as follows: i) completeness: of the 14 health facilities visited, only 6 (42.9%) supplied all the information needed for the surveillance system. Regrettably, most of the eight health facilities (57.1%) did not provide all of the requested data, which raises questions regarding the accuracy and completeness of the surveillance data gathered. ii) Timeliness: of the 14 health facilities, only 5 (35.7%) met the deadlines for providing data to the surveillance system. Nonetheless, a troubling majority of 9 healthcare facilities (64.3%) failed to achieve the necessary timeliness requirements (Figure 3). Accuracy: according to the investigation, six (42.9%) of the 14 health facilities showed good accuracy in the data they collected for surveillance. However, a worrying majority of 8 health facilities (57.1%) had low accuracy, indicating a significant need for process improvement in the data gathering. The authenticity and dependability of the surveillance data gathered from these sites are called into doubt by this disparity in accuracy levels 4. Surveillance utilization: the results show that only one health institution (7.1%) made effective use of the surveillance data when making decisions and taking action. On the other hand, 92.9% of the 13 health facilities surveyed did not effectively utilize the surveillance information that was gathered. The surveillance system's potential benefits are hampered by its under-utilization, which prevents it from informing and directing public health actions and programs.

In this evaluation, 22 healthcare professionals and 12 health extension workers took part. Nineteen (86%) of the 22 health professionals were male, while all the health extension workers were females. The interquartile range (IQR) for health professionals' ages was 25-35 years, with 26 being the median. Health Extension Workers had an average age of 25 years (interquartile range: 22-28 years). Regarding the background of the health workers, 7 (32%) were health officers, and 15 (68%) were nurses. Health professionals and health extension personnel had an average of 5 and 8 years of experience, respectively. The frequency of perinatal deaths documented per expected estimation is modest, with 13 reported in the facility examined during system evaluation whereas maternal deaths were sixteen. Health facilities, hospitals, and health posts stored weekly surveillance reports in file cabinets. While hospitals and facilities had the Maternal and Perinatal Death Surveillance and Response manual, not all health posts did. Among the 14 visited health facilities, only one (17%) had a maternal death notification form. Additionally, one health center (40%), one hospital (100%), and one health post (17%) reported cases of maternal death verbal autopsy (Table 4). The evaluation gathered feedback from both healthcare professionals and health extension workers on various aspects of a surveillance system. The results indicated differing opinions on simplicity, flexibility, acceptance, sensitivity, positive predictive value, Representativeness, timeliness, cost, and usefulness. While some expressed satisfaction, others were dissatisfied with different aspects of the system. The findings suggest a need for further research to address specific areas of improvement based on the varying opinions expressed (Table 5).

**Table 4:** availability of reporting and recording of surveillance system in Meda Welabu Woreda Bale Zone, 2023

SN	Variables		Hospital (n=1)	Woreda health office (n=1)	Health center (n=6)		Health post (n=6)		Total (n=14)	
			Frequency	Frequency	Frequency	Percentage	Frequency	Percentage	Frequency	Percentage %
1	Public health emergency management national guideline	Yes	1	1	6	100	3	50	11	79
		No	0	0	0	0	3	60	3	21
2	Maternal and perinatal death surveillance system Implementation Manual	Yes	1	1	6	100	0	0	8	57
		No	0	0	0	0	6	100	6	43
3	Weekly Report formats	Yes	1	1	6	100	3	60	11	79
		No	0	0	0	0	3	40	3	21
4	Maternal death notification forms	Yes	1	NA	3	40	0	0	4	29
		No	0	0	3	60	6	100	9	64
5	Maternal verbal autopsy	Yes	0	1	1	40	1	20	3	21
		No	0	0	5	60	5	80	10	71
6	Facility based maternal death abstraction form	Yes	1	0	2	40	NA	NA	3	21
		No	0	0	4	60	NA	NA	4	29
7	Case-based maternal death reporting formats	Yes	1	1	2	20	NA	NA	4	29
		No	0	0	4	80	NA	NA	4	29
8	Rumor logbook	Yes	0	1	0	0	0	0	1	7
		No	0	0	6	100	6	100	12	86
9	Line list	Yes	1	1	3	60	0	0	5	36
		No	0	0	3	40	6	100	9	64
10	Public health emergency management case-based reporting format	Yes	1	1	3	60	NA	NA		0
		No	0	0	3	40	NA	NA		0

**Table 5:** overall surveillance system among health extension workers and health professionals, 2023

Variables		Health professionals		Health extension workers	
		Frequency	Percentage %	Frequency	Percentage %
simplicity	positive	9	41	8	67
	Negative	13	59	4	33
Flexibility	positive	13	59	6	50
	Negative	9	41	6	50
Acceptability	positive	13	59	8	67
	Negative	9	41	4	33
Sensitivity	positive	13	59	9	75
	Negative	9	41	3	25
Predictive value positive	positive	7	32	4	33
	Negative	15	68	8	67
Representativeness	positive	12	55	9	75
	Negative	10	45	3	25
Timeliness	positive	13	59	4	33
	Negative	9	41	8	67
Cost	positive	12	55	3	25
	Negative	10	45	9	75
Usefulness	positive	11	50	8	67
	Negative	11	50	4	33

## Discussion

### Simplicity

**Health professionals:** nine of the 22 health professionals surveyed gave the system a positive rating for simplicity, while 12 gave it a poor rating. Out of twelve health extension workers, eight expressed satisfaction with its simplicity, while four expressed dissatisfaction. These findings imply that the professionals surveyed had differing opinions about what constitutes simplicity. Additional research may be able to pinpoint particular areas where simplicity could be improved.

### Flexibility

**Health workers:** nine health professionals disagreed with the perception held by 13 health professionals that the system was flexible.

**Health extension workers:** six of these workers gave favorable input regarding system flexibility, while the other six had conflicting views. These findings suggest that, depending on the reactions, the system might be somewhat flexible. Attempts could be undertaken to tackle the issues brought up and enhance system adaptability even more.

### Acceptance

**Health workers:** of them, nine expressed doubts about the system, while thirteen deemed it to be acceptable.

**Health extension workers:** of these, 8 said they thought the system was acceptable, and 4 said they didn't. This suggests that while a sizable portion of experts find the system generally acceptable, there is still need for improvement to allay the worries voiced by certain individuals.

### Sensitivity

**Health workers:** of the health professionals surveyed, nine disagreed with the idea that the system is sensitive, while thirteen said it was. Health Extension Workers: Three of the workers disagreed that the system is sensitive, whereas nine felt it is. These findings show that the professionals who were interviewed had differing opinions about how sensitive the system should be. To increase the system's sensitivity and comprehend the causes of the disagreements, more research may be necessary.

### Positive predictive value, or PPV

**Health workers:** of the professionals surveyed, 7 expressed satisfaction with the PPV, while 15 expressed dissatisfaction.

**Health extension workers:** only 4 out of 80 health extension workers expressed good opinions about the PPV, with the rest (78 out of 80) having negative feedback. These findings imply that the majority of the professionals surveyed believe the surveillance system's PPV to be poor. Improving the system's performance in this area would require addressing the causes of this perception.

**Representativeness:** healthcare professionals - of the 22 healthcare professionals surveyed, 12 thought the system was representative, while 10 didn't. Twelve health extension workers were interviewed; nine of them thought the system was representative, and three did not. These findings point to a varied opinion of Representativeness among the experts surveyed. In order to determine the places where the system might not be able to obtain a representative sample, more research may be necessary.

### Timeliness

**Health workers:** of the health professionals surveyed, nine disagreed and 13 said the system delivered information in a timely manner.

**Health extension workers:** conversely, of the health extension workers, four gave favorable comments on timeliness and eight did not. These findings show some disagreement about how timely the system is thought to be. Gaining an understanding of the elements causing the variations may help improve the timeliness of the system.

### Cost

**Health workers:** ten health professionals thought the system's cost was negative, while 12 thought it was appropriate.

**Health extension employees:** of the health extension employees, three thought the system's cost was reasonable, while nine felt otherwise. These findings imply that opinions on the expense of the surveillance system are divided. In order to identify potential cost improvements, it would be beneficial to investigate the reasons influencing the negative opinions.

### Usefulness

**Health professionals:** usefulness of the health experts surveyed, 11 thought the method was useful, while the remaining 11 had mixed feelings.

**Health extension workers:** eight of these workers thought the system was helpful, whereas four did not share this opinion. These findings show that the professionals who were interviewed had differing opinions about its value. The areas where the system fails to provide healthcare practitioners with relevant information could be found with more research. The evaluation of a population's general health and well-being depends heavily on its maternal and perinatal mortality rates [7,11,12]. In order to gather and analyze data about maternal and perinatal fatalities, surveillance systems are essential [11]. With an emphasis on the accuracy of the data gathered and its application, we shall assess the results of the surveillance evaluation carried out in Meda Welabu Woreda health facilities in this discussion. According to the finding of the surveillance evaluation, performance indicates; eight cases (57%) with inadequate accuracy in the monitoring system's data collection on maternal and perinatal mortality indicated a poor level of precision, according to the study.

Addressing this issue is critical because precise statistics are needed to pinpoint problem regions and carry out efficient treatments. The review also indicated that 6 cases (42.9%) had good data collection accuracy. It's critical to acknowledge these instances of accuracy and pinpoint the elements that made them successful. According to the review, the monitoring system successfully and promptly delivered data in 5 cases (35%). Because timely intervention is essential to preventing maternal and perinatal fatalities. This result is positive because timely intervention is essential to preventing maternal and perinatal fatalities. However, the data supplied by the surveillance system was delayed in the majority of cases (64.3%). This suggests that the system's ability to transmit data quickly has to be improved. The study indicates that completeness, six cases (42.9%) had complete data in the surveillance system. Accurate data is necessary to recognize trends, risk factors, and to put the right measures in place. In contrast, the data in eight cases (57.1%) were incomplete. Inaccurate analysis and limited monitoring effectiveness in reducing maternal and perinatal death can result from incomplete data. The assessment revealed that good data quality was present in 57.1% of the data gathered, suggesting some degree of validity and consistency in the surveillance system. But in terms of validity, reliability, and consistency, 40.9% of the data were of low quality. This result emphasizes how procedures for documenting and gathering data need to be improved.

**Encounter utilization:** in surveillance system, data analysis and utilization are important issue however, there was just one instance (7.1%) where the surveillance data was used efficiently. Policymakers and healthcare providers may make well-informed decisions, carry out focused interventions, and protect the health of expectant mothers and perinatal when surveillance data is used extensively. In contrast, there was no indication of surveillance use in the vast majority of cases (92.9%). This poor utilization rate necessitates raising awareness, developing capability, and enhancing surveillance integration. Health professionals' and health extension Workers' overall MPDSR knowledge was 43% and 41%, respectively. This is less than the 50% seen in the Zimbabwe study [13]. This discrepancy may be the result of the availability of training so that the healthcare professionals of the individuals in the prior study could receive a lot of training. The increase in internet availability could serve as yet another defense. Now that we have internet access, we might read more, which might force health professionals and health extension workers to learn more about MPDSR. The woreda weekly report form had an average completion rate of 88%. This is higher than the studies done in Mutare district, Zimbabwe, 76%, and Zimbabwe, 79% [14]. Only 7 reports of maternal deaths from hospitals and Zero report of health facilities were delivered to the woreda among 16 maternal fatalities, demonstrating the low timeliness of this surveillance system. In contrast to the normal FMOH report, which is due within 48 hours, and another study carried out in Ethiopia, the case-based type of maternal mortality reported from the hospital was reported after 2 months for 9 MDSR [1].

**Limitation/data quality:** the majority of the fields in the surveillance reporting format were filled in correctly, but the following data quality gaps were found: blank spaces that should have been filled in with zero (0) numbers were not recorded, and the beginning and ending dates of the week were also not entirely filled in. Additionally, the date the report was received and transmitted is a crucial factor in determining how timely the reporting was, yet it is frequently overlooked at all healthcare facilities

## Conclusion

The surveillance evaluation system of maternal and perinatal mortality uncovered both limitation and strength. Significant opportunities for improvement were found, despite of good timeliness, completeness, accuracy, quality of data, and use. Prioritizing expanded use of the surveillance system's data should go hand in hand with improving the quality, timeliness, accuracy, and completeness of the information gathered. In the end, this will improve the general health outcomes of the people in Meda Welabu Woreda by enabling more efficient interventions and methods to lower maternal and perinatal mortality. In summary, the finding of the Maternal and Perinatal Death System Evaluation surveillance system at Meda Welabu Woreda health institutions in the Bale Zone reveals notable deficiencies in the data's timeliness, accuracy, completeness, and usage. These results show that in order to raise the overall caliber and efficacy of the surveillance system monitoring, feedback, weekly bulletin, strong committee and improvement are required.
